# The Structural and Biological Effects of Zinc and Titanium Oxide Nanoparticles on the Condition of Activated Sludge from a Municipal Wastewater Treatment Plant

**DOI:** 10.3390/ma18194523

**Published:** 2025-09-29

**Authors:** Anna Kwarciak-Kozłowska, Krzysztof Łukasz Fijałkowski

**Affiliations:** Faculty of Infrastructure and Environment, Czestochowa University of Technology, Brzeźnicka 60a, 42-200 Czestochowa, Poland; anna.kwarciak@pcz.pl

**Keywords:** nanoparticles, activated sludge, zinc oxide, titanium dioxide, toxicity, dehydrogenase activity, particle size distribution, wastewater treatment, floc fragmentation

## Abstract

The increasing environmental presence of metal oxide nanoparticles (NMOPs) raises concerns regarding their influence on biological wastewater treatment. This study comparatively evaluates the structural and biological effects of zinc oxide (ZnO-NPs) and titanium dioxide (TiO_2_-NPs) nanoparticles on activated sludge from a wastewater treatment plant. Experimental exposure covered nanoparticle concentrations of 0.05–0.3 g/L and contact times up to 180 min, with analysis of enzymatic activity (dehydrogenase activity, TTC-SA method), sludge settleability, and particle size distribution. Inhibition of microbial metabolic activity was observed in a clear dose- and time-dependent manner, with ZnO-NPs showing stronger toxicity than TiO_2_-NPs. At the highest dose (0.3 g/L), enzymatic activity nearly disappeared after 90 min (0.04 µg TPF/mg MLSS). Both nanoparticles caused floc fragmentation, decreased sludge volume index (SVI), and increased the proportion of ultrafine particles (<0.3 µm). ZnO-NPs induced more severe destabilization, while TiO_2_-NPs showed partial re-aggregation of suspended particles at higher concentrations. Additionally, particle size distribution in the supernatant was analyzed, revealing distinct aggregation and fragmentation patterns for ZnO- and TiO_2_-NPs. These structural and functional alterations suggest potential risks for treatment efficiency, including reduced nutrient removal and impaired sludge settleability. The study provides a comparative contribution to understanding toxicity mechanisms of ZnO- and TiO_2_-NPs and emphasizes the need to monitor NMOPs in wastewater and to develop mitigation strategies to ensure stable plant operation

## 1. Introduction

The rapid development of nanotechnology has raised concerns regarding the potential environmental and health impacts of nanomaterials. Their incorporation into mass consumption products, such as cosmetics, sunscreens, food additives, paints, and detergents, has led to their widespread distribution in the environment. Once released, these engineered nanoparticles (ENPs) enter wastewater systems, where they interact with microbial communities in activated sludge—the core of biological treatment processes [1,2,3,4]. Nanoparticles present in ecosystems may originate from both natural and anthropogenic sources. Natural inputs include soil erosion, sandstorms, volcanic eruptions, or forest fires, while anthropogenic sources are linked to pharmaceuticals, medical diagnostics, environmental remediation, and personal care products. According to the Project on Emerging Nanotechnologies (PEN), the most common nanoparticles in consumer products are silver (48%), titanium dioxide (10%), carbon (9%), silicon dioxide (5%), and zinc oxide (4%). Their release into municipal wastewater is well documented; for example, nanosilver-coated textiles can release measurable silver ions during washing [5,6,7].

Among metal oxide nanoparticles (NMOPs), titanium dioxide (TiO_2_-NPs) and zinc oxide (ZnO-NPs) are of particular concern. They are widely applied due to their photocatalytic and antimicrobial properties and are frequently detected in sewage sludge. Table 1 summarizes selected physicochemical characteristics of ZnO-NPs and TiO_2_-NPs, including particle size, density, solubility, and reactivity. Their small dimensions and large surface-to-volume ratio enable interaction with biomolecules, penetration of cell membranes, and the induction of oxidative stress [8,9,10]. They are classified within the broader category of nanoparticles known as Engineered NanoParticles or Nano Metal Oxide Particles (NMOPs). Six specific NMOPs have been identified in sewage sludge: ZnO-NPs, TiO_2_-NPs, CuO-NPs, CeO_2_-NPs, MgO-NPs, and MnO_2_-NPs [6].

They are classified within the broader category of nanoparticles known as Engineered NanoParticles (ENPs) or Nano Metal Oxide Particles (NMOP_s_). Six specific NMOPs have been identified in sewage sludge: ZnO-NPs, TiO_2_-NPs, CuO-NPs, CeO_2_-NPs, MgO-NPs, and MnO_2_-NPs [6].

Compared to nanoparticles of the same category of elements, zinc oxide nanoparticles (ZnO-NPs) exhibit increased effectiveness against Gram-positive bacteria [8]. The properties of zinc and titanium nanoparticles depend primarily on their dimensions, composition, crystal structure, and shape. Changing their size to the nanoscale level can potentially modify their chemical, mechanical, electrical, structural, morphological, and optical properties. These tailored properties enable nanoparticles to bind characteristically with biomolecules at the cellular level, thereby promoting the physical translocation of nanoparticles into internal cellular components. Nanostructured materials exhibit a higher proportion of atoms on their surface, resulting in increased surface reactivity [11,12].

NMOPs aggregate and precipitate in the aquatic environment under the influence of electrostatic and van der Waals forces [10]. Aggregation and precipitation processes have a significant impact on the change in the form and level of exposure of NMOPs in aqueous solutions, with the existing form and concentration in water serving as indicators of their degree of exposure. For example, in water, TiO_2_ nanoparticles occur in the form of 10–100 nanoaggregates, while ZnO nanoparticles occur in the form of 250–400, 500–950, and 500–1150 nanoaggregates in seawater, respectively [6,13,14,15].

NMOPs with characteristic physicochemical properties can effectively penetrate the cell membrane of microorganisms and enter the internal environment of the cell. The interaction between NMOP_s_ and cells causes depolarization of the cell membrane, liquefaction, and increased passive permeability. As a result, essential intracellular components, including ions, adenosine triphosphate (ATP), nucleic acids, sugars, enzymes, and amino acids, leak out, leading to a transport imbalance, disruption of respiratory pathways, interruption of energy transfer, cell dissolution, and ultimately, cell death [6,14,16].

The decisive factors influencing their toxicity are physicochemical properties, such as pore structure, specific surface area, and surface hydrophilicity. TiO_2_ in the anatase phase is characterized by a large specific surface area and superhydrophilicity under UV radiation, which enhances reactive oxygen species generation and promotes interactions with EPS and microbial cell surfaces. ZnO, on the other hand, exhibits lower surface stability but higher solubility, leading to Zn^2+^ release as the dominant toxicity pathway. Its moderately hydrophilic surface promotes aggregation in complex aqueous matrices, which at higher concentrations may reduce the bioavailability of the particles to microorganisms. Although both nanoparticles are considered effective antibacterial agents, their environmental safety in complex biological systems, such as activated sludge, remains under evaluation [16,17]. Zinc oxide nanoparticles are considered a safe bactericidal substance that exhibits a broad spectrum of activity against various strains of bacteria, as verified by numerous researchers.

Studies conducted using high-throughput sequencing revealed changes in microbial biodiversity with increasing concentrations of TiO_2_-NPSs (0–60 mg/L) in reactors. These changes indicated a correlation between toxicity and nanoparticle concentration. The toxic effect was associated with increased production of reactive oxygen species (ROS) and lactate dehydrogenase (LDH), which can disrupt the integrity of the microbial membrane [1,17].

ZnO-NPs and TiO_2_-NPs nanoparticles are used in a wide range of products, which results in their introduction into sewage systems, through which they ultimately reach wastewater treatment plants. Currently, NMOPs are identified as one of the rapidly emerging pollutants at municipal wastewater treatment plants [2,5,18,19,20].

NMOPs can potentially threaten the safe and stable operation of activated sludge systems. Little is known about how and to what extent NMOPs affect the performance of sludge systems. Therefore, the impact of NMOP stress on overall performance, microbial communities, and metabolic characteristics has become a topic of growing interest worldwide [6,17,18].

Biological wastewater treatment relies on diverse microbial consortia to degrade organic matter and remove nutrients. The presence of NMOPs in treatment systems has been shown to affect microbial activity, enzymatic pathways, and floc stability, with consequences for settleability and nutrient removal efficiency. While several studies have investigated nanoparticle impacts on overall treatment performance, fewer have addressed their influence on microfaunal indices such as the Biotic Index of Activated Sludge (IBIO) or physical parameters such as the sludge volume index (SVI). Moreover, the structural effects of NMOPs on sludge flocs and their release of particles into the supernatant remain poorly documented [19,20,21,22].

It has been found that NMOPs inhibit microbial activity and exhibit ecological toxicity to aquatic organisms [5]. The effect of NMOP on activated sludge systems was investigated, and it was found that its toxic effect is related to the dose, type, and duration of exposure [22]. The high photocatalytic activity of NMOPs causes significant damage to the microbial environment in activated sludge [6,23,24].

Since wastewater contains more than one type of nanoparticle, it is necessary to understand the interaction of both single and mixtures of two or more nanoparticles on bacteria to quantitatively determine their subsequent impact on the performance of biological reactors [25].

In terms of studying the fate of nanoparticles in conventional activated sludge systems, it has been found that nanoparticles typically adsorb onto activated sludge flocs and then penetrate the interior of microbial cells [1,26]. The limited data published in this field are contradictory. In the case of microparticles (MPs), there have been almost no published data to date on their impact on the activated sludge system. As for nanoparticles, it has been found that ZnO–NPs improve the sedimentation properties of activated sludge; however, the size of the flocs decreased or increased depending on the composition of the wastewater [1,27].

Considering that the wastewater treatment system functions as a dynamic entity, it is crucial to comprehend its reactions when subjected to NMOP nanoparticles. While numerous researchers are investigating this area, there remains a scarcity of information regarding the effects of a combination of nanoparticles (both in the short term and long term) on activated sludge characteristics [25].

Most existing studies focus on the effects of nanoparticles on the biological performance of activated sludge, particularly regarding the removal of nitrogen, phosphorus, and organic carbon. However, limited attention has been given to their impact on the physical structure of sludge flocs and the potential release of particles into the supernatant.

Hypothesis and objectives: We hypothesize that ZnO-NPs exert stronger toxic and structural effects on activated sludge than TiO_2_-NPs, leading to greater reductions in enzymatic activity, microfaunal diversity, and floc stability. Therefore, this study aims to (i) compare the effects of ZnO- and TiO_2_-NPs on enzymatic activity (DHA), sludge microfauna (IBIO), settleability and SVI, and particle size distribution; (ii) identify dose-dependent thresholds of toxicity; and (iii) assess the implications of these effects for the stability of biological wastewater treatment processes.

## 2. Materials and Methods

### 2.1. Research Substrate

The substrate for the study was activated sludge obtained from a municipal wastewater treatment plant (conventional) serving a population equivalent (PE) of 242,081. The activated sludge in the biological wastewater treatment chambers was continuously mixed and aerated with compressed air. The pH of the activated sludge was 6.72. The total dry matter content of the activated sludge used was 3.28 g/dm^3^, which corresponds to a typical concentration for biological digesters operating stably under aerobic conditions. The post-calcination residue, constituting the mineral fraction of the sludge, was 2.12 g/dm^3^, while the organic dry matter content reached 1.16 g/dm^3^. The organic-to-mineral fraction ratio indicates the high biological activity of the sludge and the presence of a significant number of microorganisms involved in biochemical treatment processes. These parameters confirm that the sludge used came from a system operating under stable conditions. Samples (1 L) were taken from the aeration chamber, transported in refrigerated containers and analyzed within 6 h.

The study utilized two types of nanoparticles, both from Sigma-Aldrich (Saint Louis, MO, USA), in the form of nanopowder: titanium dioxide (P25) and zinc oxide. TiO_2_ is a fibrous, odorless, white nanopowder with a particle size of less than 100 nm, a molecular weight of 79.87 g/mol, and a relative density of 4.17 g/cm^3^. Zinc oxide, like TiO_2_, was in the form of a white nanopowder with a particle size of less than 100 nm; however, it had a higher molecular weight (81.41 g/mol) and density (5.68 g/cm^3^).

**Figure 1:**
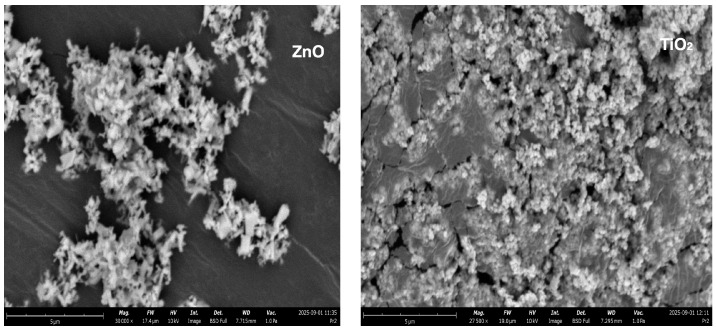
The SEM image of ZnO and TiO_2_.

SEM image (Phenom ProX G6, Thermo Fisher Scientific, Eindhoven, The Netherlands) (Scanning Electron Microscope) analysis and EDS spectroscopy data enabled detailed morphological and chemical characterization of the zinc oxide (ZnO) and titanium oxide (TiO_2_) nanoparticles used in the study. In the case of ZnO, the SEM image revealed the presence of numerous nanoparticle clusters with a distinctly irregular crystalline structure (Figure 1). These particles exhibit sharp edges and a diverse morphology, including rod-like, needle-like, and tabular forms—typical of the hexagonal wurtzite crystal system. The nanoparticles form irregularly shaped secondary aggregates resembling branched colonies. Based on the scale used (8 µm), the primary units were estimated to be approximately 150–300 nm in size, secondary aggregates were estimated to be 1–3 µm in size, and single crystals were approximately 200–500 nm thick. The SEM image of TiO_2_ indicates a distinct surface structure. The nanoparticles exhibit a spherical or hemispherical morphology and form a densely packed, porous layer with a characteristic granular texture. Primary units are difficult to distinguish due to the dense agglomeration of the particles. This type of microstructure may indicate the presence of anatase or brookite phases—known for their high specific surface area and widespread use in photocatalytic processes. To confirm the chemical composition of both materials, EDS analysis was performed in conjunction with SEM. The ZnO sample was found to contain two dominant elements: oxygen (O) and zinc (Zn). The atomic content was 29.94% (O) and 13.46% (Zn), respectively, and the weight content was 22.70% (O) and 41.70% (Zn), respectively. After normalization of the atomic and weight values, the following were obtained: 68.99% (O) and 31.01% (Zn)—on an atomic basis, and 35.25% (O) and 64.75% (Zn)—on a weight basis, respectively. These proportions are consistent with the theoretical stoichiometric composition of ZnO, confirming the high purity of the tested material. For the TiO_2_ sample, EDS analysis also revealed the presence of two key elements: oxygen (O) and titanium (Ti). The atomic content was 43.76% (O) and 6.87% (Ti), while the weight content was 41.50% (O) and 19.50% (Ti). After normalization, the following values were obtained: 86.43% (O) and 13.57% (Ti) for the atomic composition, and 68.03% (O) and 31.97% (Ti) for the weight composition. In both cases, the chemical composition obtained in EDS studies is consistent with literature data for pure metal oxides, confirming the quality of the nanomaterials used.

### 2.2. Analytical Methods

To assess the impact of nanoparticles on the activated sludge biocoenosis, observations were carried out using an Olympus BX41 light microscope (Olympus, Tokyo, Japan). The biological quality of the sludge was assessed based on the Biotic Index for Activated Sludge (IBIO), which considers the presence and structure of microorganisms colonizing the activated sludge. Sludge activity was determined using the HACH Lange method (LCK 318 cuvette test) by DIN 38412-3 [28] using the TTC-SA (Merck, Darmstadt, Germany) (Triphenyl Tetrazolium Chloride—Substrate Activity) test. This test allowed the assessment of changes in the metabolic activity of microorganisms under the influence of nanoparticles. Determination of sludge activity with 2,3,5 2,3,5-triphenyltetrazolium chloride (TTC) based on dehydrogenase activity. TTC is converted to red formazan by dehydrogenases. The water-insoluble formazan is extracted with ethanol and determined photometrically according to:

Sludge activity As = (μg formazan TF)/(mg mixed liquor suspended solids MLSS) = C1/C2(1)

C1—measurement result,

C2—V × TS; V = 4.3 mL,

TS—Mixed liquor suspended solids MSLL [mg/L],

As—Activity of the sludge expressed in μg formazan, represented by 1 mg MSLL.

The dry mass (DM), dry organic mass (DOM) and mineral matter (MM) were determined in the tested activated sludge in accordance with the PN-EN 12879 [29].

Characterization of the ZnO and TiO_2_ nanoparticles involved an assessment of their surface morphology and elemental composition, as determined by scanning electron microscopy (SEM) and energy-dispersive X-ray spectroscopy (EDS) (Phenom ProX G6, Thermo Fisher Scientific, Eindhoven, The Netherlands). Before imaging, the nanoparticle powders were immobilized on a dedicated holder and coated with a ~10 nm layer of gold using a Leica EM ACE200 sputter coater (Leica Microsystems CMS GmbH, Wetzlar, Germany), operating under low-vacuum conditions. Morphological observations were conducted using a scanning electron microscope (Phenom ProX, Thermo Fisher Scientific, Waltham, MA, USA), while the elemental composition was determined via an integrated EDS detector.

### 2.3. Experimental Setup

**Figure 2:**
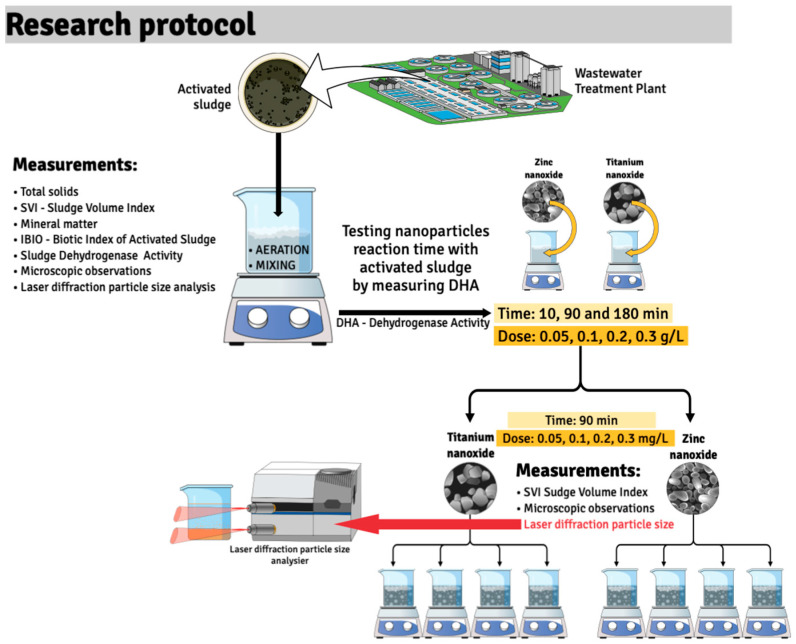
Research protocol.

The experiment was conducted in 300 mL glass reactors (Chemland, Stargard, Poland), into which the collected activated sludge was introduced (Figure 2). The experiment was carried out under natural daylight conditions, which was intended to reproduce the actual conditions in biological reactors in municipal wastewater treatment plants. All measurements were taken in triplicate.

An Air Pump 400 (EHEIM, Deizisau, Germany) with a power of 4 W and an adjustable capacity of up to 400 L/h was used to aerate the reactor contents. The system was equipped with a fine-bubble aeration stone connected to the pump, which allowed a stable dissolved oxygen (DO) concentration of ≥2.0 mg/L to be maintained, corresponding to the conditions in a classic oxygen chamber of a wastewater treatment plant. The air flow rate was manually adjusted using a throttle valve, which allowed for the appropriate aeration intensity while maintaining the integrity of the activated sludge floc structure.

In the first step of the study, the characteristics of the activated sludge collected from the municipal treatment plant were determined (microscopic observation, IBIO index, sludge settleability, dry sludge mass, and organic and mineral content).

To assess the impact of zinc and titanium nanoparticles on activated sludge, they were added to the reactors directly with continuous mixing in doses of 0.05 g/L, 0.1 g/L, 0.2 g/L, and 0.3 g/L. The study assessed the impact of nanoparticles on the sludge settleability, sludge volume index, its metabolic activity (TTC-SA), and the distribution of nanoparticles in activated sludge and supernatant. The decomposition of nanoparticles in the tested samples was measured using the Analysette 22 Next Nano laser particle analyzer (FRITSCH, Idar-Oberstein, Germany). This type of laser particle analyzer allows for the examination of solid particles, suspensions, and wet emulsions in the range of 0.01 to 3800 μm.

## 3. Results

### 3.1. Biological Assessment of Activated Sludge Based on the IBIO Index

To assess the biological quality of activated sludge, the Biotic Index of Activated Sludge (IBIO) was used, based on microscopic analysis of the microfauna present in the sample (Table 1).

The identification parameters included the dominant group of organisms, microfauna density, number of taxonomic units, and abundance of small flagellates (denoted as F). The analyzed sample was dominated by amoebae and sessile and crawling ciliates, which are characteristic of well-stabilized activated sludge [30]. The microfauna density exceeded 10^6^ individuals/dm^3^, and the number of taxonomic units exceeded 10, which indicates high biological diversity. The number of small flagellates, F, was less than 10, which further confirms the maturity of the sediment [31]. Based on the above parameters, according to the IBIO assessment table, the IBIO index value was determined to be 10, indicating an excellent biological quality of the activated sludge. This result suggests that the system is operating under proper aerobic conditions, is stable, and exhibits high efficiency in the biological treatment process. No signs of overload, destabilization, or the presence of toxic substances adversely affecting the microfauna of the sludge were found.

### 3.2. Determination of Reaction Time and DHA Activity of Activated Sludge

Scientific reports primarily focus on the use of nanoparticle doses ranging from 1 to 100 mg/L [19,32,33,34], although a few studies suggest the need to assess the impact of higher concentrations, even above 200 mg/L [24]. Most studies, as in the present work, focus on their short-term impact on activated sludge microflora. Short-term exposure allows for the capture of early biological reactions of the sludge, such as changes in enzymatic activity or changes in sludge settleability and the SVI parameter. The results shown in Table 2 indicate that dehydrogenase activity in activated sludge decreases systematically with increasing TiO_2_ nanoparticle concentration and increasing contact time.

Under control conditions (0 g/L TiO_2_), the enzymatic activity remains relatively stable, regardless of the reaction time (4.58–4.68 μg TPF/mg MLSS). For the higher doses (0.2 and 0.3 g/L), potent DHA inhibition is observed as early as 15 min, worsening after 90 min, and continuing or slightly decreasing after 180 min. For the highest dose (0.3 g/L), enzymatic activity almost completely disappears after just 90 min (0.04 μg TPF/mg MLSS). Similarly to TiO_2_, ZnO nanoparticles exhibit an apparent toxic effect on activated sludge microorganisms, as evidenced by a decrease in dehydrogenase activity (Table 3).

The toxic impact of ZnO-NPs is particularly evident at doses ≥ 0.1 g/L and with prolonged exposure. At the highest dose (0.3 g/L), DHA activity decreases from 0.85 μg TPF/mg MLSS after 15 min to 0 after 180 min, showing a similar pattern to that of TiO_2_ nanoparticles. Nevertheless, ZnO-NPs exhibit a slightly stronger inhibitory effect at the same doses and exposure times than TiO_2_-NPs, which may result from the different specificity of the interaction of zinc nanoparticles with microorganism cells. Based on the results, a reaction time of 90 min was chosen as the optimal measurement point for further toxicity analyses of titanium-zinc oxide nanoparticles. This time represents a compromise between early exposure (15 min), where toxic effects may not yet be fully expressed, and a longer contact time (180 min), where enzymatic activity for high doses virtually disappears, making it difficult to differentiate effects between samples. After 90 min, clear dose-dependent differences in DHA levels are observed, which allows the precise determination of toxicity thresholds and response mechanisms of sediment microorganisms to the presence of nanoparticles. Exposure to increasing concentrations of TiO_2_ nanoparticles resulted in a progressive suppression of DHA. At the highest dose (0.3 g/L), activity nearly disappeared after just 90 min of contact, indicating a strong cytotoxic effect. These findings are consistent with literature reports showing that TiO_2_ nanoparticles can interfere with enzymatic systems through multiple mechanisms, primarily by generating reactive oxygen species (ROS) and disrupting extracellular polymeric substances (EPS), which leads to impaired microbial functionality [35]. ZnO nanoparticles also caused substantial reductions in DHA, with observable effects beginning as early as 15 min after exposure at ≥0.1 g/L. The complete inhibition of activity at 0.3 g/L by 180 min further confirms their potent toxicity. This stronger effect, when compared with TiO_2_-NPs, aligns with other studies reporting that ZnO nanoparticles not only induce ROS but also dissolve to release Zn^2+^ ions, which are known to bind cellular thiol groups, disrupt membrane transport, and inhibit enzymatic activity at the intracellular level [35,36]. Importantly, the observed suppression of DHA at higher nanoparticle doses could be attributed to both oxidative and non-oxidative stress responses. Mahmood et al. [36] showed that both protein levels and DHA in activated sludge decline significantly upon nanoparticle exposure, indicating cell lysis or metabolic inhibition. These changes were evident within the first 0.5–3 h of exposure, corroborating the inhibitory dynamics observed in our 15–180 min experimental window. ZnO-NPs consistently showed stronger inhibition than TiO_2_-NPs at equivalent concentrations and exposure times, suggesting differences in interaction specificity with microbial cells. While TiO_2_-NPs induced toxicity may sometimes be partially reversible at lower concentrations, ZnO-NPs effects tend to be more acute and persistent, as confirmed by the complete inactivation of DHA at higher doses. At 90 min of exposure, DHA inhibition was significantly stronger in the presence of ZnO-NPs (~85%) compared to TiO_2_-NPs (~60%), confirming the higher toxicity of ZnO-NPs under the studied conditions. Such strong inhibition of enzymatic activity may considerably limit nutrient removal efficiency in real wastewater treatment plants, highlighting the potential operational risks associated with the presence of metal oxide nanoparticles.

### 3.3. Determination of Sludge Settleability and Sludge Volume Index (SVI)

The conducted research revealed clear differences in the impact of TiO_2_-NPs and ZnO-NPs on the sedimentation properties of activated sludge, specifically in terms of sludge settleability (mL/L) and sludge volume index (SVI, mL/g).

For the control sample of activated sludge without the addition of nanoparticles, these values were 480 mL/L (settling rate) and 146.3 mL/g (SVI). The results obtained indicate that the sludge collected from the municipal wastewater treatment plant exhibited good biological activity; however, its increased sedimentation volume may suggest the onset of swelling processes [27,37]. The typical SVI range for properly functioning sludge is 50–150 mL/g. Values above 150 mL/g may indicate sludge bulking, often associated with excessive growth of filamentous bacteria or disruption of the EPS structure. Values below 50 mL/g are defined as heavy sludge, settling very well, but may indicate limited biological activity, the presence of toxic substances, or excessive sludge loading.

**Figure 3:**
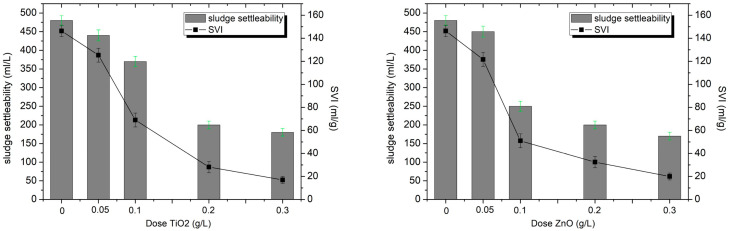
The effect of ZnO- and TiO_2_-NPs doses on sediment settleability and SVI.

It was observed that with increasing TiO_2_-NPs doses, the settleability and SVI gradually decreased, reaching a level of 180 mL/L at the highest dose used (0.3 g/L) and an SVI of only 17 mL/g. In the case of ZnO-NPs, a significantly stronger destabilizing effect on the activated sludge was observed, with settleability reaching 170 mL/L (dose 0.3 g/L). The greatest differences between the two oxides were observed at a dose of 0.1 g/L. The settling index for ZnO-NPs and TiO_2_-NPs reached 250 mL/L and 370 cm^3^/L, respectively, and the SVI was 51 mL/g (ZnO-NPs) and 69 mL/g (TiO_2_-NPs) (Figure 3).

This suggests that ZnO-NPs have a stronger effect on the structure of activated sludge flocs and on the composition of extracellular polymeric substances (EPS), which are responsible for sludge cohesion and sedimentation. According to studies conducted [38,39], ZnO nanoparticles exhibit greater toxicity to microorganisms than TiO_2_ Nanoparticles. This is related, among other things, to the generation of Zn^2+^ ions and reactive oxygen species (ROS), which contributes to the decomposition of EPS and reduce the ability of the sediment to form compact aggregates, resulting in smaller floc volumes and faster settling. This phenomenon was also observed in the work of Jinyu Ye et al. [40], who demonstrated a correlation between EPS damage and a decrease in SVI, in real plant conditions, the long-term presence of nanoparticles may destabilize sludge settling processes, posing a risk to the operational stability of wastewater treatment systems. In the case of TiO_2_, despite the sedimentation effect also being observed, this impact is milder, which may be related to its lower solubility and reduced accessibility to the internal structure of the flocs. From the perspective of wastewater treatment plant operation, such a low SVI index may be desirable due to improved sludge thickening and dewatering efficiency. However, it should be remembered that excessive destabilization of the floc structure can negatively affect biological processes such as nitrification, denitrification, and BOD removal. The impact of ZnO nanoparticles on wastewater treatment efficiency is well-documented. Zheng et al. [34] demonstrated that exposure to ZnO-NPs at a concentration of 10–50 mg/L led to inhibition of enzymatic activity, including nitrite oxidoreductase and polyphosphate kinase. They observed a decrease in nitrogen removal, from 81.5% to 75.6–70.8%, and an increase in its concentration in treated wastewater (up to 10.3–16.5 mg/L), indicating a degradation in the effectiveness of biological processes in activated sludge. The destruction of activated sludge flocs may impair both denitrification and phosphorus removal processes, leading to reduced overall nutrient removal efficiency in wastewater treatment. Despite the extensive literature on the impact of ZnO-NPs on pollutant removal and microbial communities, their impact on the flocculation and sedimentation of activated sludge has been less studied to date. This study was limited to short-term experiments conducted under controlled laboratory conditions, which may not fully reflect the complexity of full-scale wastewater treatment plants. In the case of TiO_2_, partial re-aggregation of sludge flocs was observed after the initial destabilization, which suggests that the effects of nanoparticle exposure may be at least partially reversible. In contrast, ZnO nanoparticles induced a permanent destabilization of the activated sludge structure, indicating that their impact is not reversible under the studied conditions.

### 3.4. Laser Diffraction Particle Size Analysis of Activated Sludge and Supernatant Water

**Figure 4:**
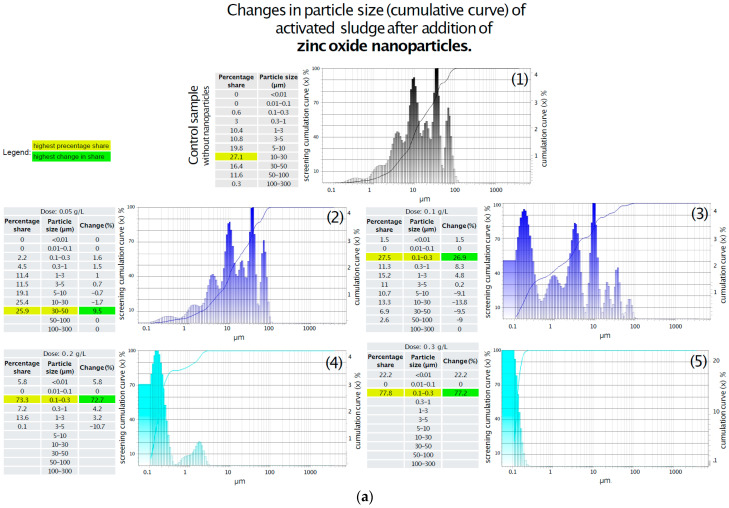

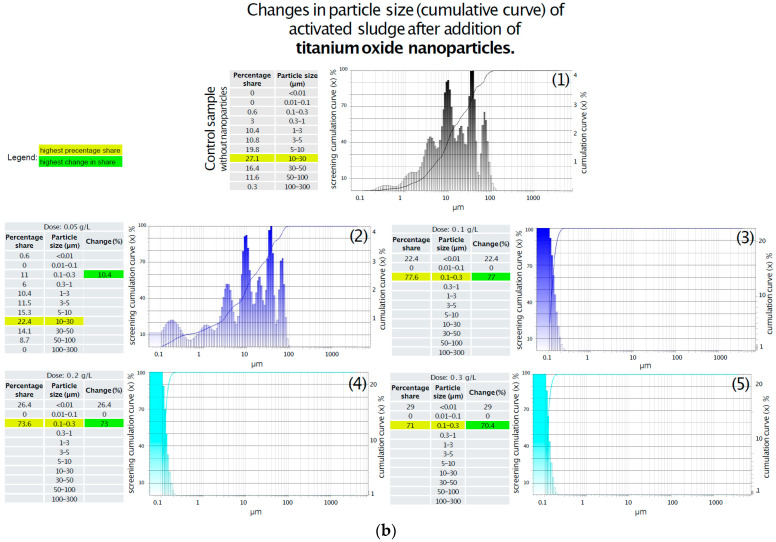
(**a**) Changes in particle size (cumulative curve) of activated sludge after the addition of ZnO-NPs. (1) Without addition of ZnO-NPs; (2) After addition of ZnO-NPs in dose 0.05 g/L; (3) After addition of ZnO-NPs in dose 0.1 g/L; (4) After addition of ZnO-NPs in dose 0.2 g/L; (5) After addition of ZnO-NPs in dose 0.3 g/L. (**b**) Changes in particle size (cumulative curve) of activated sludge after the addition of TiO_2_-NPs. (1) Without addition of TiO_2_-NPs; (2) After addition of TiO_2_-NPs in dose 0.05 g/L; (3) After addition of TiO_2_-NPs in dose 0.1 g/L; (4) After addition of TiO_2_-NPs in dose 0.2 g/L; (5) After addition of TiO_2_-NPs in dose 0.3 g/L.

Changes in activated sludge particle size distribution following the addition of titanium oxide were analyzed, and the results are summarized in Figure 4a. In the control sample, the highest proportion of particles (27%) was found in the 10–30 μm size range, while the smallest share was observed for particles smaller than 1 μm. The introduction of 0.05 g/L of titanium oxide resulted in a 10% increase in the fraction of particles sized 0.1–0.3 μm, accompanied by the fragmentation of particles larger than 5 μm; however, the dominant size range remained 10–30 μm. Increasing the dose to 0.1 g/L resulted in the severe fragmentation of flocs larger than 3 μm, with the 0.1–0.3 μm fraction becoming the dominant fraction and accounting for 77.6% of the total particle population. No significant changes were observed with further increases in the titanium oxide dose to 0.2 g/L and 0.3 g/L compared to the 0.1 g/L treatment, apart from a gradual rise in the proportion of particles smaller than 0.01 μm, which reached 22%, 26%, and 29%, respectively (Figure 4).

The results demonstrate a clear correlation between zinc oxide concentration and the fragmentation of activated sludge particles. As illustrated in Figure 4b, the addition of 0.05 g/L of zinc oxide induced minor structural changes, including a reduction in the number of particles in the 5–30 µm range and a shift toward smaller fractions between 0.1 and 5 µm. In contrast, at a concentration of 0.1 g/L, a more pronounced breakdown of particles larger than 5 µm was observed, accompanied by an increase in particles within the 0.1–5 µm range, particularly in the 0.1–0.3 µm fraction. At a dose of 0.2 g/L, a substantial fivefold increase in the tiniest particles (<0.01 µm) was recorded, along with an increase in the 0.1–0.3 µm fraction to 74%, and a severe fragmentation of flocs larger than 5 µm. The highest dose (0.3 g/L), shown in Figure 4a, resulted in even more extensive fragmentation, with the <0.01 µm fraction reaching 22% and the 0.1–0.3 µm range comprising 78% of the total particle population. Analysis of the particle size distribution in the supernatant following titanium oxide addition revealed significant changes.

**Figure 5:**
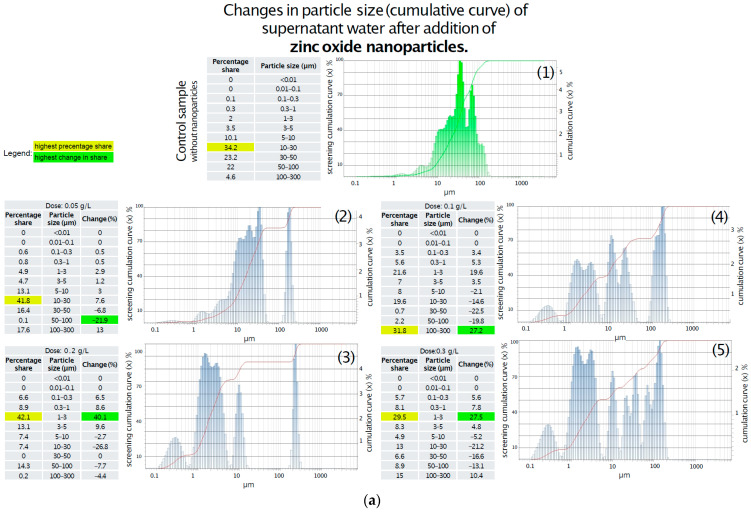

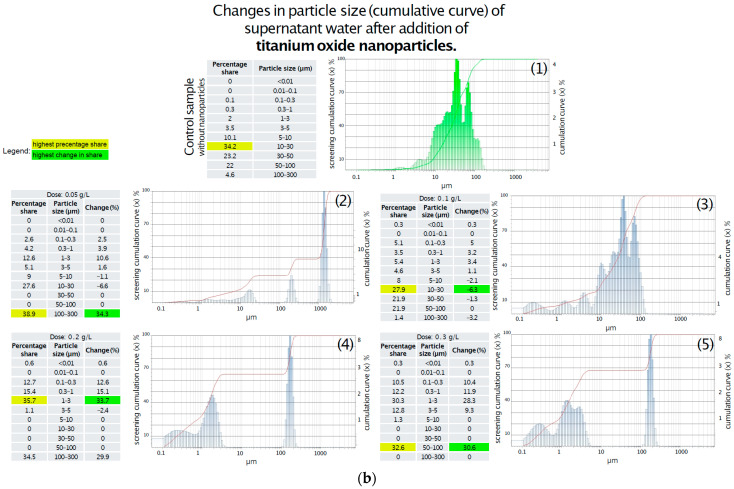
(**a**) Changes in particle size (cumulative curve) of water supernatant after the addition of various doses of ZnO-NPs. (1) Without addition of ZnO-NPs; (2) After addition of ZnO-NPs in dose 0.05 g/L; (3) After addition of ZnO-NPs in dose 0.1 g/L; (4) After addition of ZnO-NPs in dose 0.2 g/L; (5) After addition of ZnO-NPs in dose 0.3 g/L. (**b**) Changes in particle size (cumulative curve) of water supernatant after the addition of various doses of TiO_2_-NPs. (1) Without addition of TiO_2_-NPs; (2) After addition of TiO_2_-NPs in dose 0.05 g/L; (3) After addition of TiO_2_-NPs in dose 0.1 g/L; (4) After addition of TiO_2_-NPs in dose 0.2 g/L; (5) After addition of TiO_2_-NPs in dose 0.3 g/L.

In the control sample showed on Figure 5a, the dominant fraction consisted of particles larger than 10 µm, primarily within the 10–30 µm range, with no particles detected below 0.1 µm. The addition of 0.05 g/L of titanium oxide led to a modest 3% increase in the 0.1–1 µm fraction, while the 10–30 µm range remained dominant. At a concentration of 0.1 g/L, a slight increase in particles < 5 µm was observed, including a 0.3% presence of particles smaller than 0.01 µm; however, the 10–30 µm fraction still prevailed. The 0.2 g/L dose caused fragmentation of larger particles (>5 µm), primarily generating particles in the 1–3 µm range, with the <0.01 µm fraction doubling to 0.6%. Notably, at the highest concentration of 0.3 g/L, a 10% increase in the 0.01–1 µm range was recorded, along with the emergence of a distinct fraction of large particles (50–100 µm), comprising over 32% of the total. This suggests a complex interaction between titanium oxide and sediment particles, possibly involving aggregation phenomena. Further analysis of sediment particle size in the supernatant following zinc oxide addition revealed dose-dependent structural changes. As shown in Figure 5b, the addition of 0.05 g/L of zinc oxide led to significant degradation of large particles (30–100 µm), with a ~20% reduction in this fraction and a minor increase in particles < 10 µm. Upon increasing the dose to 0.1 g/L, particles in the 5–30 µm range underwent further fragmentation, accompanied by a 20% increase in the 1–3 µm fraction, while the 100–300 µm fraction became dominant. The application of 0.2 g/L of zinc oxide resulted in a 40% increase in the 1–3 µm range and a moderate (7%) increase in smaller particles. At the highest dose of 0.3 g, no substantial differences were noted compared to the 0.2 g/L treatment, with the 1–3 µm fraction remaining dominant and a slight decrease observed in particles > 10 µm.

A summary of the changes in particle size is presented below:

A. Effect of TiO_2_-NPs on activated sludge:
  1. In the control sample, the dominant particle size was within the 10–30 μm range (27%).
  2. The addition of 0.05 g/L TiO_2_-NPs resulted in a 10% increase in particles sized 0.1–0.3 μm and fragmentation of larger particles (>5 μm), while the 10–30 μm range remained dominant.
  3. At 0.1 g/L TiO_2_, complete fragmentation of particles > 3 μm was observed, with 77.6% of particles falling within the 0.1–0.3 μm range.
  4. No significant changes were observed at 0.2 g/L and 0.3 g/L compared to 0.1 g/L, apart from a gradual increase in particles < 0.01 μm (22%, 26%, and 29%, respectively).
B. Effect of ZnO-NPs on activated sludge:
  1. A clear dose-dependent relationship was observed between ZnO concentration and particle fragmentation.
  2. The addition of 0.05 g/L ZnO-NPs slightly reduced the number of particles in the 5–30 μm range and increased the proportion of particles sized 0.1–5 μm.
  3. At 0.1 g/L, more intense fragmentation of particles > 5 μm was observed, increasing the share of 0.1–5 μm particles.
  4. A dose of 0.2 g/L resulted in a fivefold increase in particles < 0.01 μm and an increase in the 0.1–0.3 μm fraction to 74%.
  5. At 0.3 g, further fragmentation was observed, with 22% of particles < 0.01 μm and 78% within the 0.1–0.3 μm range.
C. Particle size distribution in supernatant water after TiO_2_-NPs addition:
  1. The control sample was dominated by particles > 10 μm, particularly in the 10–30 μm range.
  2. The addition of 0.05 g/L TiO_2_-NPs resulted in a minor (3%) increase in particles sized 0.1–1 μm, with larger particles still predominant.
  3. At 0.1 g/L, a slight increase in particles < 5 μm and the appearance of a 0.3% fraction < 0.01 μm were observed.
  4. The 0.2 g/L dose caused fragmentation of larger particles (>5 μm), generating more 1–3 μm particles and doubling the <0.01 μm fraction to 0.6%.
  5. At 0.3 g/L, a 10% increase in the 0.01–1 μm fraction was noted, along with the appearance of a significant 50–100 μm fraction (over 32%), suggesting complex aggregation or restructuring processes.
D. Particle size distribution in supernatant water after ZnO-NPs addition:
  1. The addition of 0.05 g ZnO-NPs reduced the quantity of large particles (30–100 μm) by ~20%, with a slight increase in smaller fractions.
  2. Increasing the dose to 0.1 g/L resulted in fragmentation of 5–30 μm particles and a 20% increase in the 1–3 μm range.
  3. At 0.2 g/L, a 40% increase in 1–3 μm particles was observed, along with an average 7% increase in smaller fractions.
  4. The highest dose (0.3 g/L) produced no significant changes relative to 8 g/L; the 1–3 μm fraction remained dominant, with a slight reduction in particles > 10 μm.

Both titanium dioxide and zinc oxide significantly influence the particle size distribution of activated sludge and supernatant water. The observed effects are dose-dependent and differ in intensity and direction depending on the type of oxide used, indicating distinct mechanisms of interaction with sludge flocs and suspended solids. Future studies should also examine the combined effects of mixed nanoparticle types, as these are more representative of real wastewater conditions and may lead to synergistic or antagonistic interactions.

#### Particle Size Analysis of Activated Sludge and Supernatant Water—Critical Review

The addition of titanium dioxide nanoparticles (TiO_2_-NPs) and zinc oxide nanoparticles (ZnO-NPs) to activated sludge resulted in significant structural changes in floc morphology and particle size distribution. The observed effects were dose-dependent and varied in intensity depending on the type of metal oxide used (Table 4).

In the case of TiO_2_-NPs, even at a low dose of 0.05 g/L, partial fragmentation of particles > 5 μm and an increase in the 0.1–0.3 μm fraction were observed. At 0.1 g/L, complete breakdown of flocs larger than 3 μm occurred, with the 0.1–0.3 μm fraction becoming dominant (77.6%). Further increases in TiO_2_-NPs concentration (0.2–0.3 g/L) led to a progressive accumulation of particles < 0.01 μm, suggesting surface erosion and destabilization of extracellular polymeric substances (EPS). These findings are consistent with the results of Jinyu Ye et al. [40], who demonstrated that exposure to TiO_2_-NPs at 100 mg/L significantly inhibits biological nitrogen removal and disrupts microbial community structure and floc integrity. ZnO-NPs exhibited a markedly more destructive impact on activated sludge. At just 0.1 g/L, fragmentation of particles > 5 μm intensified, while particles < 0.3 μm became dominant. At doses of 0.2 and 0.3 g/L, the <0.01 μm fraction increased fivefold (up to 29%), and floc structure was almost entirely disintegrated. These effects corroborate the findings of Liu et al. [41], who attributed the toxicity of ZnO-NPs to both zinc ion release (Zn^2+^) and oxidative stress. Meli et al. [42] further confirmed that ZnO-NPs significantly reduce the diversity and abundance of key microbial taxa in activated sludge, particularly those involved in nitrification. Analysis of the water supernatant revealed distinct nanoparticle-induced shifts in particle distribution. For TiO_2_-NPs, a high dose (0.3 g/L) led to the emergence of both ultra-fine particles (<0.01 μm) and unexpectedly large aggregates (50–100 μm), which may indicate nanoparticle-induced re-aggregation or restructuring of EPS. Similar phenomena were described by Brar et al. [18], who observed complex interactions between metal oxide nanoparticles and organic matter, leading to floc formation or destabilization. In contrast, ZnO-NPs induced continuous fragmentation in the supernatant, without secondary aggregation. The dominant fraction shifted toward 1–3 μm at higher doses, with a gradual reduction in larger particles (>10 μm), indicating a more destructive and less selective mechanism of action. Both TiO_2_-NPs and ZnO-NPs significantly affect the physical structure of activated sludge. ZnO-NPs exhibit a more pronounced destructive impact, rapidly degrading floc architecture and increasing the presence of fine and ultrafine particles. These structural changes may negatively affect sludge settleability, floc stability, and overall process performance in biological wastewater treatment systems. The observed particle-size shifts, especially the emergence of <0.01 μm fractions, may lead to reduced sedimentation rates, and impaired biological activity. Further studies should focus on how these structural disruptions affect biofilm formation, permeability, and long-term reactor performance.

## 4. Conclusions

### 4.1. Comparison of ZnO-NPs and TiO_2_-NPs Effect on Activated Sludge

**Figure 6:**
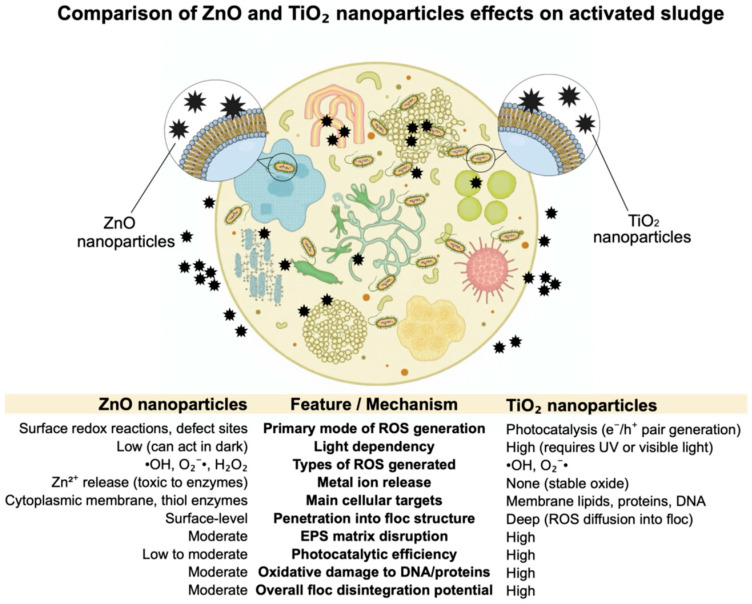
Comparison of ZnO-NPs and TiO_2_-NPs effect on activated sludge. References: [5,22,43,44,45,46].

Metal oxide nanoparticles, particularly zinc oxide and titanium dioxide, have demonstrated strong oxidative effects against organic matter, including the microbial biomass comprising activated sludge flocs (Figure 6).

These flocs consist of densely packed microbial cells embedded within an extracellular polymeric substance (EPS) matrix, composed of proteins, polysaccharides, nucleic acids, and lipids, which ensures floc integrity and functionality in wastewater treatment systems. ZnO-NPs induce oxidative stress through multiple mechanisms: generation of reactive oxygen species (ROS), electrostatic membrane disruption, and the release of Zn^2+^ ions. These ROS—such as hydroxyl radicals (•OH), superoxide anions (O_2_^−^•), and hydrogen peroxide (H_2_O_2_)—oxidize cellular components and compromise membrane integrity. Additionally, Zn^2+^ ions can bind to thiol groups in glycolytic enzymes, leading to enzymatic inhibition and metabolic dysfunction [10,43,44,47,48].

In contrast, TiO_2_-NPs, especially those composed of anatase-rutile mixtures, exhibit markedly stronger oxidative effects due to their photocatalytic capabilities. Under UV irradiation (λ ≤ 385 nm), TiO_2_ absorbs photons and generates electron-hole pairs that initiate redox reactions at the nanoparticle surface. These ROS penetrate bacterial membranes, degrade EPS components, and disrupt the structural cohesion of the flocs. Moreover, TiO_2_-NPs can depolarize bacterial outer membranes, target lipopolysaccharides and membrane proteins, and damage DNA and intracellular enzymes, further contributing to cell lysis [33,44,45].

Comparative studies highlight that TiO_2_-NPs are more effective than ZnO-NPs in degrading activated sludge structures, primarily due to higher ROS generation, deeper penetration into biofilms, and light-activated mechanisms. The photocatalytic activity of TiO_2_-NPs can be enhanced by factors such as particle morphology, UV power, and the presence of light-absorbing organic molecules. Consequently, TiO_2_-NPs represent a more aggressive oxidative agent with greater potential to destabilize activated sludge flocs, offering valuable applications in advanced oxidation processes and nanotechnology-assisted wastewater treatment systems [5,14,46,49].

This study offers critical insights into the dose-dependent effects of zinc oxide nanoparticles (ZnO-NPs) and titanium dioxide nanoparticles (TiO_2_-NPs) on the biological activity and physical structure of activated sludge, a crucial component of municipal wastewater treatment. The findings underscore the potential risks posed by these emerging contaminants to the stability and efficiency of biological wastewater treatment processes.

### 4.2. Structural Alterations and Floc Fragmentation

Both types of nanoparticles significantly altered the physical structure of activated sludge flocs, leading to fragmentation and an increase in the proportion of smaller particles. This destabilization of floc architecture can negatively impact sludge settleability and overall process performance. ZnO-NPs had a more pronounced destructive impact on floc morphology, causing more intense fragmentation and a greater increase in ultrafine particles compared to TiO_2_-NPs. This is attributed to the greater toxicity of ZnO-NPs and their ability to disrupt extracellular polymeric substances (EPS), which are crucial for floc cohesion. TiO_2_-NPs, while also causing fragmentation, showed some evidence of re-aggregation at higher doses in the supernatant, indicating complex interactions with suspended solids.

### 4.3. Implications for Wastewater Treatment

The observed reductions in DHA and significant floc fragmentation highlight the potential for impaired biological activity and reduced settling rates in activated sludge systems exposed to these nanoparticles. Such effects can lead to decreased treatment efficiency, and challenges in sludge dewaterability. The study highlights that the impact of metal oxide nanoparticles on activated sludge is dose-dependent and varies significantly between nanoparticle types, underscoring the need for further research into their long-term effects and the development of effective mitigation strategies to ensure the sustainable operation of wastewater treatment plants.

In conclusion, the presence of NMOPs represents a potential threat to the sustainability of biological wastewater treatment, as their accumulation may undermine the long-term stability and efficiency of activated sludge processes. Understanding their differential impacts on microbial activity and floc structure is crucial for developing robust wastewater treatment technologies that can handle these emerging pollutants.

These findings highlight the urgent need to establish clear regulatory frameworks for monitoring and managing metal oxide nanoparticles in wastewater, since current legislation (e.g., REACH, ECHA) does not provide specific discharge limits for these emerging contaminants.

Main conclusions from the conducted research:

1. Comparative toxicity: Both ZnO-NPs and TiO_2_-NPs negatively affected the biological and structural stability of activated sludge, with ZnO-NPs exerting stronger toxicity. Enzymatic inhibition (DHA), reductions in microfaunal diversity (IBIO), and severe floc fragmentation were most pronounced under ZnO exposure.
2. Operational implications: The observed changes in sludge settleability and enzymatic activity suggest potential risks for nutrient removal efficiency and effluent quality in wastewater treatment plants. Therefore, regular monitoring of nanoparticle concentrations in influent, effluent, and activated sludge is recommended to ensure stable process operation under real plant conditions.
3. Future perspectives: The results emphasize the importance of developing strategies to mitigate nanoparticle effects, including advanced treatment systems, such as membrane filtration or adsorption, may mitigate the entry of nanoparticles into biological reactors.

Long-term studies under real operating conditions are required, and regulatory frameworks should establish guidelines for the monitoring and management of NMOPs in wastewater.

## Figures and Tables

**Table 1:** Microfauna parameters and IBIO value of the examined activated sludge sample.

Dominant Group and Microfauna Density Determining the Order		Total Number of Taxonomic Units Forming the Microfauna of Activated Sludge and Abundance of Small Flagellates F Counted in the Belt							
Dominant key group	Total organisms in L	>10		8–10		5–7		<5	
		F < 10 10 < F < 100		F < 10 10 < F < 100		F < 10 10 < F < 100		F < 10 10 < F < 100	
House amoebas, sessile and crawling ciliates	10^6^	10	8	9	7	8	6	7	5
	<10^6^	9	7	8	6	7	5	6	4
Sedimentary >80%	10^6^	9	7	8	6	7	6	6	4
	<10^6^	8	6	7	5	6	4	5	3
*Opercularia* spp.	10^6^	7	5	6	4	5	3	4	2
	<10^6^	6	4	5	3	4	2	3	1
*Vorticella* *microstoma*	10^6^	6	3	5	3	4	2	3	1
	<10^6^	5	3	4	2	3	1	2	0
Freely swimming bacteriophagous ciliates	10^6^	5	3	4	2	3	1	2	0
	<10^6^	4	2	3	1	2	0	1	0

**Table 2:** Dehydrogenase activity (DHA) in activated sludge in the presence of titanium oxide nanoparticles as a function of reaction time and nanoparticle doses (0.05, 0.1, 0.2 and 0.3 g/L).

Reaction Time [min]	Titanium Oxide Nanoparticle Dose [g/L]				
	0	0.05	0.1	0.2	0.3
	DHA Activity [µg TPF/mg MLSS]				
15	4.58 ± 0.06	4.31 ± 0.09	2.21 ± 0.03	1.49 ± 0.04	1.03 ± 0.09
90	4.68 ± 0.06	4.46 ± 0.08	1.93 ± 0.02	0.77 ± 0.09	0.04 ± 0.03
180	4.39 ± 0.07	3.9 ± 0.06	1.04 ± 0.03	0.37 ± 0.03	0 ± 0.000

**Table 3:** Dehydrogenase activity (DHA) in activated sludge in the presence of zinc oxide nanoparticles as a function of reaction time and nanoparticle doses (0.05, 0.1, 0.2 and 0.3 g/L).

Reaction Time [min]	Zinc Oxide Nanoparticle Dose [g/L]				
	0	0.05	0.1	0.2	0.3
	DHA Activity [μg TPF/mg MLSS]				
15	4.58 ± 0.09	3.63 ± 0.08	2.01 ± 0.04	1.24 ± 0.07	0.85 ± 0.06
90	4.48 ± 0.05	3.35 ± 0.10	1.37 ± 0.07	0.18 ± 0.08	0.1 ± 0.02
180	4.39 ± 0.11	3.17 ± 0.09	0.87 ± 0.05	0 ± 0.000	0 ± 0.000

**Table 4:** Comparison: Effects of TiO_2_-NPs and ZnO-NPs on activated sludge and supernatant water.

Section A: Effects on Activated Sludge		
Parameter	TiO_2_-NPs	ZnO-NPs
Threshold dose for visible sludge disintegration	0.1 g/L	0.1 g/L
Dominant particle size after 0.1 g/L dose	0.1–0.3 μm (77.6%)	0.1–0.3 μm (74%)
Increase in ultrafine particles (<0.01 μm)	Gradual: 22–29% for 0.2–0.3 g/L	Strong: fivefold increase at 0.2 g/L
Residual large particles (>5 μm) at 0.3 g/L	Absent	Absent
Particle destabilization mechanism	Surface erosion, EPS interference, ROS generation	Zn^2+^ release, oxidative stress, cell membrane disruption
Effect on microbial diversity	Moderate, dose-dependent	Strong reduction in diversity and abundance
Observed in the literature	Yes [39,40]	Yes
**Section B: Effects on supernatant water**		
Parameter	TiO_2_-NPs	ZnO-NPs
Secondary aggregation	Yes—50–100 μm particles at 0.3 g/L (32%)	No
Peak in 1–3 μm fraction	Moderate increase at 0.2 g/L	Strong increase (40%) at 0.2 g/L
Dominant change at high doses	Emergence of large and ultrafine particles	Fragmentation toward small and medium sizes
Presence of particles < 0.01 μm	Appeared from 0.1 g/L, increased with dose	Significant from 0.2 g/L
Observed in the literature	Yes [39,40]	Yes [41,42]
References: [1,19,35,40,41,42]		

## Data Availability

The original contributions presented in this study are included in the article. Further inquiries can be directed to the corresponding author.
